# Targeted therapies in retinoblastoma: GD2-directed immunotherapy following autologous stem cell transplantation and evaluation of alternative target B7-H3

**DOI:** 10.1007/s00262-023-03587-0

**Published:** 2024-01-19

**Authors:** Thomas Eichholz, Florian Heubach, Anne-Marie Arendt, Christian Seitz, Ines B. Brecht, Martin Ebinger, Tim Flaadt, Daniela Süsskind, Lisa Richter, Isabel Hülsenbeck, Leonie Zerweck, Sophia Göricke, Frank Paulsen, Frank Dombrowski, Christian Flotho, Stefan Schönberger, Petra Ketteler, Johannes Schulte, Peter Lang

**Affiliations:** 1 University Children’s Hospital, Eberhard Karls University, Abteilung I, Hoppe-Seyler-Str. 1, 72076 Tuebingen, Germany; 2 Department of Ophthalmology, Eberhard Karls University, Tuebingen, Germany; 3 Department of Pediatrics III, University Children’s Hospital Essen, University of Duisburg-Essen, Essen, Germany; 4 Department of Ophthalmology, University Hospital Essen, University of Duisburg-Essen, Essen, Germany; 5 Department of Radiology, Diagnostic and Interventional Neuroradiology, Eberhard Karls University, Tuebingen, Germany; 6 Department of Diagnostic and Interventional Radiology and Neuroradiology, University Hospital Essen, University of Duisburg-Essen, Essen, Germany; 7 Department of Radiation Oncology, Eberhard Karls University, Tuebingen, Germany; 8 Institute of Pathology, University Medicine of Greifswald, Greifswald, Germany; 9 Division of Pediatric Hematology and Oncology, Department of Pediatric and Adolescent Medicine, Medical Center, Faculty of Medicine, University of Freiburg, Freiburg, Germany; 10 RB-Registry, University Children’s Hospital Essen, University of Duisburg-Essen, Essen, Germany; 11 Cluster of Excellence iFIT (EXC2180) “Image-Guided and Functionally Instructed Tumor Therapies”, University of Tübingen, Tuebingen, Germany; 12 German Cancer Consortium (DKTK) and German Cancer Research Center (DKFZ), Partner Site Tübingen, University of Tuebingen, Tuebingen, Germany

**Keywords:** Retinoblastoma, Targeted therapy, Dinutuximab beta, GD2, CD276, B7-H3

## Abstract

**Background:**

GD2-directed immunotherapy is highly effective in the treatment of high-risk neuroblastoma (NB), and might be an interesting target also in other high-risk tumors.

**Methods:**

The German-Austrian Retinoblastoma Registry, Essen, was searched for patients, who were treated with anti-GD2 monoclonal antibody (mAb) dinutuximab beta (Db) in order to evaluate toxicity, response and outcome in these patients. Additionally, we evaluated anti-GD2 antibody-dependent cell-mediated cytotoxicity (ADCC) and complement-dependent cytotoxicity (CDC) in retinoblastoma cell lines in vitro. Furthermore, in vitro cytotoxicity assays directed against B7-H3 (CD276), a new identified potential target in RB, were performed.

**Results:**

We identified four patients with relapsed stage IV retinoblastoma, who were treated with Db following autologous stem cell transplantation (ASCT). Two out of two evaluable patients with detectable tumors responded to immunotherapy. One of these and another patient who received immunotherapy without residual disease relapsed 10 and 12 months after start of Db. The other patients remained in remission until last follow-up 26 and 45 months, respectively. In vitro*,* significant lysis of RB cell lines by ADCC and CDC with samples from patients and healthy donors and anti-GD2 and anti-CD276-mAbs were demonstrated.

**Conclusion:**

Anti-GD2-directed immunotherapy represents an additional therapeutic option in high-risk metastasized RB. Moreover, CD276 is another target of interest.

## Introduction

Retinoblastoma (RB) is the most common malignant eye-tumor in children [1]. Stage-adjusted therapy strategies ranging from local therapies, systemic chemotherapy, radiation to high-dose chemotherapy with autologous stem cell transplantation (ASCT) result in cure rates as high as 95% [2, 3]. In localized stages, the eye or even vision can be preserved, whereas management of advanced local or metastasized disease remains challenging [4, 5]. Interestingly the disialoganglioside GD2 and B7-H3 may represent potential therapeutic targets in these tumors:

- Retinoblastoma, like neuroblastoma cells express GD2 on their surface [6, 7]. Targeting GD2 with mAbs improves survival in neuroblastoma [8–10] and is standard of care in high-risk, relapsed or refractory disease [11, 12]. Anti-GD2 antibodies [13] and anti-GD2 chimeric antigen receptor (CAR)-T cells [14, 15] mediated effective cytolysis of retinoblastoma cell lines in vitro.
- B7-H3 (CD276) functions as immune checkpoint and is found to be significantly overexpressed on a broad range of solid tumors including retinoblastoma [16]. B7-H3 is evaluated preclinically as alternative target in GD2-negative neuroblastoma with encouraging results [17].

Individual treatment attempts using Anti-GD2 mAb in patients with advanced retinoblastoma were undertaken by different treatment centers [18, personal communication]. We here describe a cohort of four patients with metastatic relapse of retinoblastoma, who were treated with the Anti-GD2 monoclonal antibody Dinutuximab beta (ch14.18/CHO) following autologous stem cell transplantation in an individual compassionate use attempt in three centers in Germany.

## Materials and methods

### Patients

The RB-registry collects data of retinoblastoma patients from Germany and Austria. The registry (initial RB diagnosis after 2013) and the hospital database (initial diagnosis prior to 2013) in Essen were searched for patients treated with Db. Clinical data of these patients on history, stage, treatment details and follow-up were obtained pseudonymized from the Retinoblastoma Registry and respective treatment centers. Informed consent was obtained from the legal representatives. The RB-registry was approved by the ethical committee, University of Duisburg-Essen (approval number 13-5405-BO). Principles stated in the declaration of Helsinki were followed. Staging followed the International Retinoblastoma Staging System (IRSS) [19].

### Pathology

GD2 expression was evaluated by different institutions and protocols. In patient 2 and 3 GD2 status was examined by the institute of pathology Greifswald immunohistochemically as described in detail earlier [20]. FFPE embedded resected metastasis temporo-basal was used in patient 2, the primary tumor of the left eye and the subsequent CNS metastasis in patient 3, respectively. In patient 1, GD2 expression was shown by immunocytology (neuroblastoma reference laboratory, Cologne) from bone marrow aspirate (EDTA). The method has been described earlier in detail [21]. GD2 status in patient 4 was evaluated on FFPE embedded resected chiasma opticus by immunofluorescence (institute of pathology Muenster).

After patient 3 relapsed following anti-GD2-directed treatment, expression of GD2 as well as B7-H3 was evaluated on resected CNS-metastasis. Evaluation of B7-H3 was done immunohistochemically by the department for pathology and neuropathology, Tuebingen. After deparaffinization and conditioning of tissue slides were incubated with CD276 (Clone: RBT-B7H3, Medac/BioSB, Wedel, Germany), diluted 1:75 for 32 min at 37 °C.

### Cell lines

RB cell lines WERI-Rb-1 and Y-79 were purchased from the German Collection of Microorganisms and Cell Cultures (DSMZ). Retinoblastoma cell lines WERI-Rb-1 and RBL-30 [22] were provided by the German RB Registry, Essen.

### Flow cytometry

Expression of GD2 and B7-H3 on RB cell lines was examined using GD2-PE (Clone 14G2a) and CD276 (Clone MIH42)-APC mAbs (Biolegend).

### In vitro ADCC/CDC cytotoxicity assays

Serum and peripheral blood mononuclear cells (PBMC) of patient 3 were used to conduct the cytotoxicity assays. Target cells WERI-Rb-1 or RBL-30 were labeled with Calcein-AM (Biolegend). Cytotoxicity mediated by effector cells (unmanipulated PBMC) and anti-GD2 (1µg/ml, Dinutuximab beta), fully humanized anti-B7-H3 (1µg/ml, supplied by Sorento Therapeutics) or patient’s serum previously treated with Dinutuximab beta (collected at *d* + 1 of the 5th mAb cycle, 12 h after last mAb infusion) was determined by calcein release after 2h. Effector cells: Fresh or frozen patient PBMCs and PBMCs from a healthy donor. Prior to use, cells were cultured overnight in RPMI (Gibco) supplemented with 10% AB Serum (Sigma). Effector to target ratio (E:T) varied between 30:1 and 40:1. This ratio has proven to be the optimal range to test mAb-mediated cytotoxicity using PBMCs of both healthy donors and patients when establishing the cytotoxicity assays locally. Cytotoxicity was calculated as the percentage of specific lysis [= (experimental calcein release—spontaneous calcein release)/(maximum calcein release–spontaneous calcein release) × 100].

## Results

Altogether 4 patients with metastatic relapse of retinoblastoma, who were treated with Dinutuximab beta between 2018 and 2021, were identified in the RB-registry. Treating centers were Essen, Tuebingen and Freiburg. Patient details are summarized in Table 1. While patient 4 was affected by sporadic retinoblastoma without germ line alteration of RB1, the others harbored a heterozygous pathogenic variant in the RB1 gene in constitutional DNA. These alterations in constitutional DNA, which represent a predisposition for the development of Retinoblastoma, did not manifest in the families before or represent a de novo mutation, respectively, and diagnosis was made after clinical manifestation of bilateral Retinoblastoma at a young age between 4 and 15 months. No extraocular, intracranial and metastatic disease was found at initial presentation.

**Table 1:** Patient Characteristics

	Patient 1	Patient 2	Patient 3	Patient 4
Treatment center	University children’s hospital Essen	University children’s hospital Essen	University children’s hospital Tübingen	University children’s hospital Freiburg
Age at diagnosis	15 months	4 months	10 months	51 months
Sex	Male	Male	Male	Male
Initial extend	Bilateral	Bilateral	Bilateral	Unilateral left eye
Constitutional RB1 alteration	del RB1 Exon 20–CHCL1 (3´ gene) Heterozygote	IVS6 + 1G > C Heterozygote	del(13)(q14.2)(RB1-) Heterozygote	No
Therapy	Enucleation RE Cryotherapy LE Systemic CT* Brachytherapy LE IVC LE IAC LE Systemic CT* Radiotherapy (proton) LE 50 Gy Resection metastasis paravertebral Systemic CT** HD + ASCT Radiotherapy (proton) 50 Gy mandibula, 46 Gy paravertebral	Systemic CT Enucleation RE Radiotherapy (photon) Systemic CT* Cryotherapy LE IAC LE Enucleation LE Systemic CT CWS**** § *Radiotherapy midface & lymph nodes (proton) 50,4 Gy §* *Systemic maintenance CT §* Systemic CT **/*** HD + ASCT Radiotherapy (proton) 44,2 Gy craniospinal	systemic CT* IAC RE IAC & IVC LE cryotherapy RE IAC RE IAC LE enucleation RE radiotherapy (proton) LE 50 Gy systemic CT*** enucleation LE systemic CT** HD + ASCT	Enucleation LE Systemic CT* Radiotherapy (proton) left orbital, N. opticus, chiasm 39 Gy exenteratio orbitae systemic CT** HD + ASCT radiotherapy (proton) 45 Gy N. opticus
Pathology enucleated eyes	Right: IRSS I	Right: not known Left: IRSS I	Right: IRSS I; N0, C2, S1. Left: IRSS I; N1, C2, S2	Left: IRSS III, N3, C2, S1
Location of metastasis (before ASCT)	IRSS Stage IVa: para-vertebral with intraspinal epidural and neuro-foraminal infiltration Th7/8, left mandibula and bone marrow infiltration	IRSS Stage IVb: right temporo-basal dura mater and soft tissue	IRSS Stage IVa paraspinal	IRSS Stage IVb: relapse infiltrating N. opticus, expanding prechiasmal
Time to metastasized disease	90 months	117 months	33 months	15 months
Confirmation of RB metastasis	Yes resection paravertebral	Yes biopsy Temporo-basal	No, biopsy not conclusive	Yes
GD2-expression	Bone marrow infiltration	Metastasis temporo-basal	Primary tumor, LE	Metastasis, chiasma opticus
Remission status prior anti-GD2 mAb	Residual tumor left mandibula	Complete remission	Residual tumor left orbital	Complete remission
Time to anti-GD2 mAb treatment after ASCT	5 months	4 months	54 days	5 months
Number and dose of anti-GD2 mAb	5 cycles á 10d 100 mg/m^2^	3 cycles á 10d 100 mg/m^2^ *¥*	5 cycles á 5d 100 mg/m^2^	5 cycles á 10d 100 mg/m^2^
Response to anti-GD2 mAb	Yes	Not assessable	Yes	Not assessable
Relapse from start anti-GD2 mAb	No	No	Yes CNS left frontal GD2 negative, B7H3 positive 12 months	Yes leptomeningeal 10 months
Last follow-up from start anti-GD2 mAb	45 months	26 months	Death 18 months	11 months
Accompanying disease	No	Alveolar rhabdomyosarcoma	No	Seizure Stenosis left A. carotis int. and occlusion left A. cerebri media, ant, post CNS abscess encephalocele left frontal Intraventricular bleeding

*LE* left eye, *RE* right eye, *CT* chemotherapy, *IVC* intravitreal chemotherapy, *IAC* intraarterial chemotherapy, *HD + ASCT* high dose chemotherapy and autologous stem cell transplantation

*systemic chemotherapy according RB Registry

**systemic chemotherapy according NB 2004-HR

***systemic chemotherapy, TECC–scheme according CWS Guidance

****systemic chemotherapy according CWS Guidance, subgroup H for very high-risk aRMS

§therapy for aRMS, ¥ first cycle 8d, therapy stopped at 3rd cycle

Despite stage-adjusted therapy, all patients suffered from one or more relapse or progression, respectively, resulting eventually in metastasized Stage IV disease according IRSS. Multimodal therapy in all patients consisted of two to six treatment lines, including at least two different schemes of systemic chemotherapy, enucleation and radiotherapy. In patients with bilateral disease eye-preserving treatment of at least one eye was attempted with local therapies like cryocoagulation, brachytherapy, selective intraarterial chemotherapy and intravitreal chemotherapy. Nevertheless, sequential enucleation of the second eye was inevitable in two patients. Finally, following rescue therapy all patients received consolidating high-dose chemotherapy with subsequent ASCT.

**Fig. 1:**
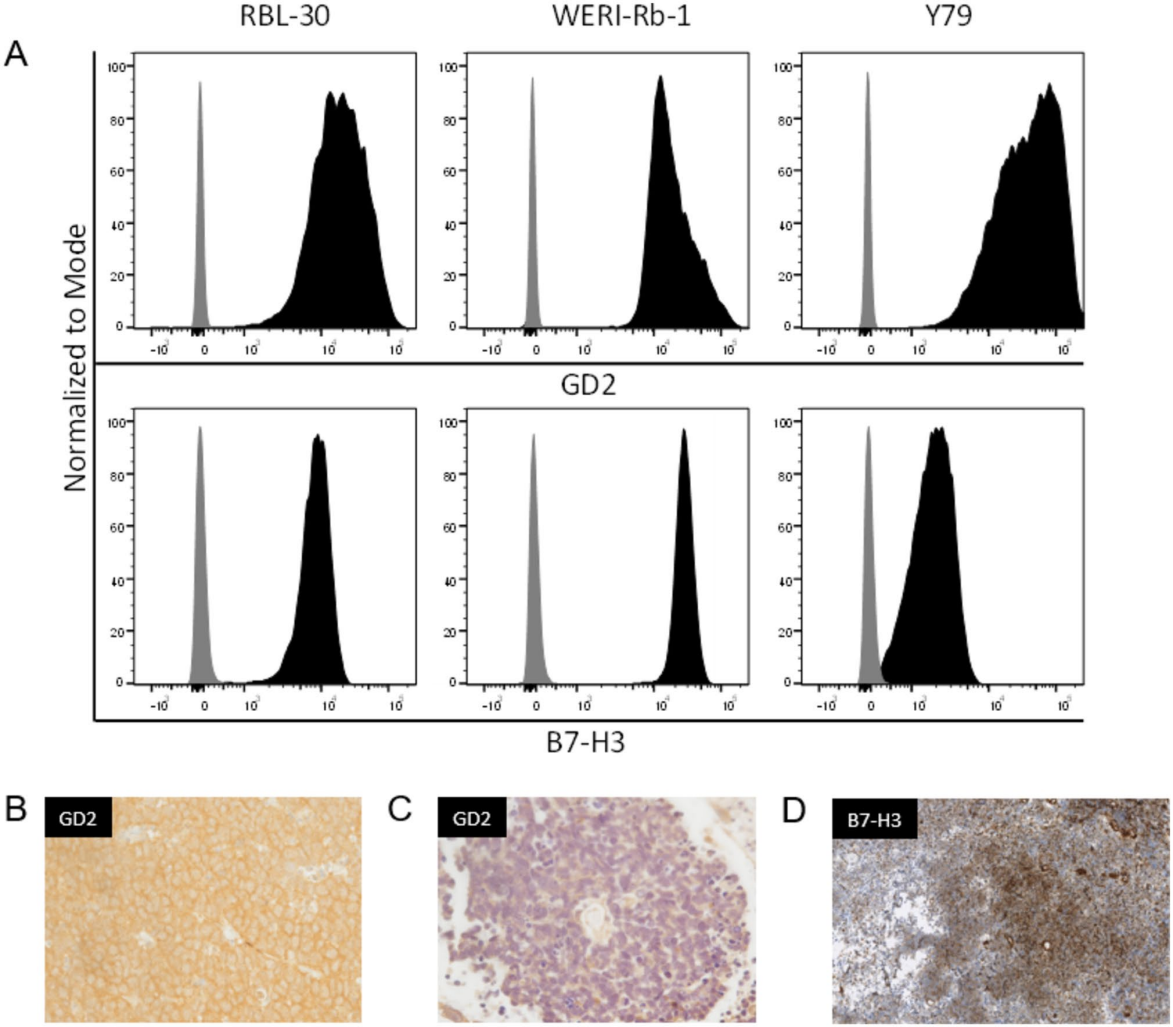
GD-2 and B7-H3 expression. **A** GD-2 and B7-H3 expression on retinoblastoma cell lines. **B** Prior to anti-GD2 mAb therapy: resected tumor sample shows membranous and intracytoplasmic GD2 expression. **C** After anti-GD2 therapy: resected tumor sample at relapse does not longer express GD2. **D** B7-H3 expression on tumor sample at relapse

The risk of renewed recurrence was considered to be high in all of the reported patients by the respective treating center. Evaluation of tumor tissue from the primary tumor or metastasis for therapeutic targets revealed an expression of GD2 on tumor cells (Fig. 1B). Therefore, analogous to high-risk NB protocols, 5 cycles of post-consolidation immunotherapy with Dinutuximab beta were applied on a compassionate use basis after informed consent for off-label therapy. Dose per cycle was 100mg/m^2^ over 5 days (patient 3) or 10 days (patients 1, 2, 4), respectively, according to treatment centers standard every 5 weeks. Supportive medication was given as described [23] including Morphine, Pregabalin or Gabapentin following local standards.

While treatment with mAb Dinutuximab beta was tolerated without serious side effects in patient 1 and 4, the first antibody cycle had to be interrupted in patient 3 due to capillary leakage with respiratory insufficiency on the second day. Symptoms resolved fast and the course was recommenced with a lower infusion rate. Subsequent courses were tolerated satisfiable.

Side effects in patient 2 were more profound with respiratory insufficiency, capillary leakage, arterial hypotony, urticaria and bronchial obstruction. The first cycle was reduced by 2 of 10 days, the second had to be interrupted. Eventually therapy had to be permanently stopped after anaphylactic reaction at the third cycle.

**Fig. 2:**
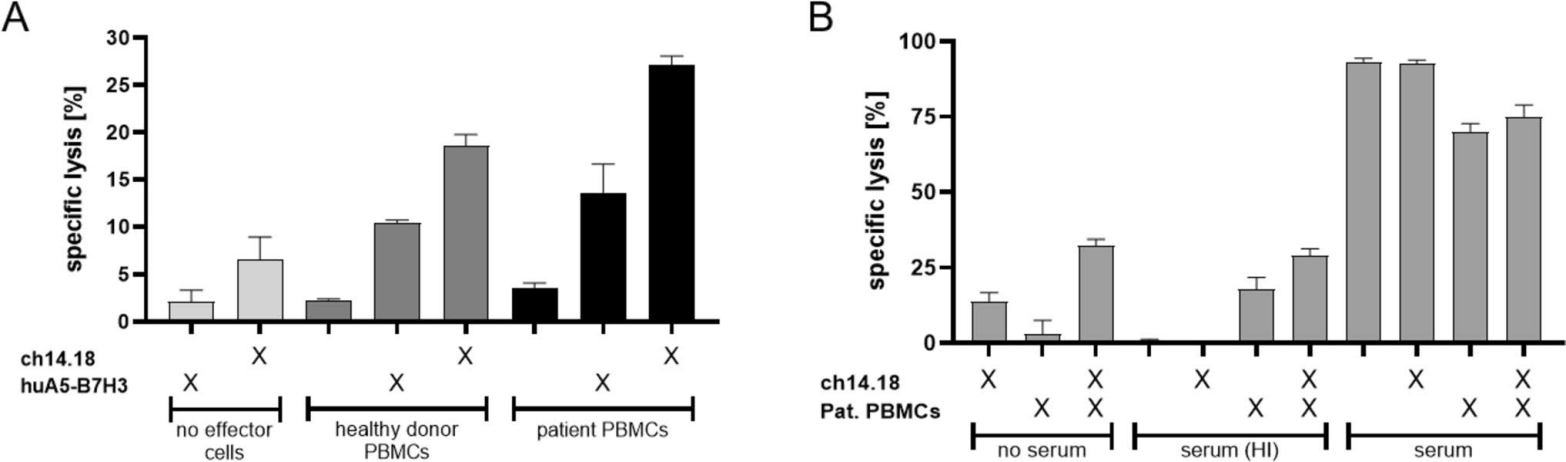
Patient ‘s PBMCs and autologous serum mediate ADCC and CDC against retinoblastoma cell lines WERI-Rb-1 and RBL-30 in 2h calcein release assays. Mean + SD of three technical replicates. **A** RBL-30 were incubated with fresh patient´s PBMCs or PBMCs from a healthy control donor (E:T = 30:1) either alone or in combination with 1 µg/ml anti-GD2 mAb ch14.18/CHO or anti-B7-H3 mAb huA5-B7-H3. The low-grade cytolysis seen in control without target cells might be explained by residual complement in the reaction medium in terms of a CDC. **B** WERI-Rb-1 were incubated as indicated with previously frozen patient´s PBMCs (E:T 40:1) and/or autologous serum alone or in combination with ch14.18/CHO to assess ADCC and CDC. Medium or heat-inactivated (HI) serum served as controls

In vitro investigations showed high expression of GD2 on all examined RB cell lines (Fig. 1A). Cytotoxicity assays using PBMCs of patient 3, revealed their capability to specifically lyse the retinoblastoma cell line RBL-30 by ADCC when combined with ch14.18/CHO. Compared to patient’s PBMCs alone, target cell lysis was significantly increased when ch14.18/CHO was present (Fig. 2A). ch14.18/CHO main effector mechanism also relies on serum complement. Thus, we additionally tested patient’s serum for its capability to induce target cell lysis by CDC. Serum was taken 12 h after anti-GD2 mAb application on day one of the 5th cycle. Serum in combination with patient’s PBMCs but also serum alone significantly increased RB target cell lysis compared to control groups containing heat-inactivated or no serum at all serum. This clearly demonstrates the presence and the cytolytic activity of ch14.18/CHO in patient’s serum during the course of mAb therapy (Fig. 2B). Taken together, these data show the functionality of patient’s PBMCs to induce ADCC in combination with ch14.18/CHO and that mAb is contained in sufficient amounts within patient’s serum to mediate CDC in vitro.

**Fig. 3:**
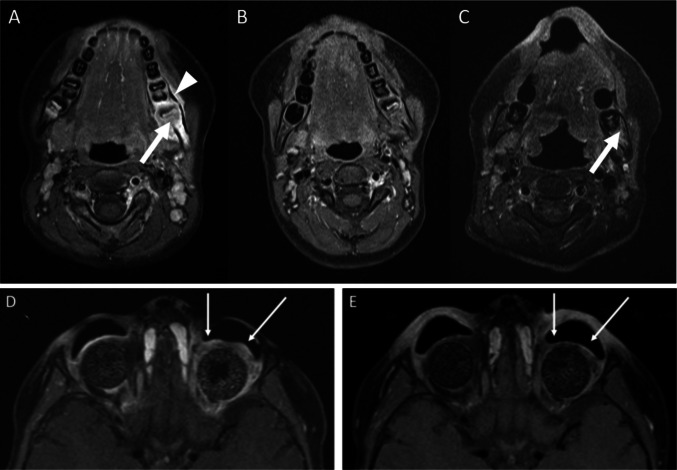
Response to Anti-GD2 therapy. **A** *Patient 1*: T1 weighted MRI with contrast agent and fatsaturation: new tumorous lesion in the left mandible (arrow) with periosseus tumor rim (arrow head). **B** *Patient 1:* imaging 2 months after ASCT, prior to RT and Dinutuximab. **C** *Patient 1:* imaging 9 months after Dinutuximab with slight residual contrast enhancement of the mandible (arrow). **D** & **E** *Patient 3*: Axial contrast-enhanced fat-saturated T_1_-weighted MR images of one bilateral enucleated 4-year-old patient with retinoblastoma before Fig. 3D and after 5 cycles of immunotherapy with Dinutuximab beta (8 months later) Fig. 3E. The image before immunotherapy reveals a residual contrast-enhanced circular manifestation of the retinoblastoma around the left orbital implant which is extended to the retrobulbar space. After antibody therapy with Dinutuximab beta the tumor remains detectable, but both, the tissue proliferation and the contrast-enhancement are distinctly regressive in the area of tumor manifestation around the orbital implant (see arrows). Extension along the optic nerve, its sheath and especially into the intracranial spaces is not present

Follow-up ranged from 11 to 45 months. Since patient 2 and 4 received Dinutuximab beta in complete remission (CR), response evaluation was restricted to the remaining two patients. In patient 1 paravertebral metastasis was no longer detectable after resection and systemic chemotherapy, whereas metastasis at the left mandibula persisted. In patient 3 the residual tumor mass in the left orbita remained after ASCT and was evaluable for further treatment response. Regression of target lesions could be observed in both patients, leading to CR in patient 1 (Fig. 3A–C) and tumor regression in patient 3 (Fig. 3D–E).

Despite initial response in patient 3 to antibody treatment, patient 3 and 4 suffered from a subsequent intracranial relapse 12 and 10 months after the start of GD-2 therapy, respectively, resulting in an Event Free Survival of 50% after 2 years. Renewed biopsy of metastasis in patient 3 showed a loss of GD2 expression (Fig. 1B–C). Instead, a strong expression of B7-H3 was found (Fig. 1D). B7-H3 is known to be expressed on a platitude of solid tumor and a clear expression could also be confirmed on all of our evaluated RB cell lines (Fig. 1A). Compared to GD-2, B7-H3 is likewise expressed on cell lines RBL-30 and WERI-RB-1. In case of Y79 though, B7-H3 is expressed on a rather low level when compared to GD-2. Using a fully humanized anti-B7-H3 mAb (huA5-B7-H3), patient’s PBMCs and PBMCs from a healthy donor mediated effective cytolysis against RBL-30 which, however, was less pronounced when compared to ch14.18 (Fig. 2A). As huA5-B7-H3 is not yet approved at the stage of a new relapse, palliative radiotherapy was applied and the patient deceased six months later.

In patient 2, an alveolar Rhabdomyosarcoma (aRMS) developed 5 years after diagnosis of bilateral retinoblastoma, but before its metastatic relapse occurred. Initial treatment of retinoblastoma in this patient consisted of systemic chemotherapy, photon irradiation of the left eye in Schipper technique and sequential enucleation of the right and later the left eye. The aRMS was localized at right Os zygomaticum/Maxilla [N+, M0], assumably in close proximity of the previous irradiation field. The aRMS was successful treated according corresponding protocols with chemotherapy, irradiation and resection.

## Discussion

Experience in treatment of neuroblastoma with Dinutuximab beta draw interest in this therapy against other tumors expressing GD2, among others retinoblastoma [7]. By the time of our studies, immunotherapy using anti-GD2 antibodies in retinoblastoma was reported simultaneously by other treatment centers [18]. Two studies using I131-labeled Anti-GD2 antibody 3F8 in GD2 positive tumors permitted, among other entities, the inclusion of patients with retinoblastoma [24, 25]. To our knowledge, there are currently no prospective studies above this, evaluating anti-GD2 mAb in retinoblastoma specifically. The rarity of high-risk stage IV retinoblastoma impedes the realization of such studies.

Using patient´s PBMCs and serum, an antibody-dependent cell-mediated cytotoxicity against GD2-positive RB cell lines could be confirmed in vitro*.* Beside this, a strong complement-dependent cytotoxicity was induced by patient´s serum alone. Since patient´s PBMCs and serum were taken during the last treatment cycle, this indicates relevant levels of mAb in patient’s serum, the absence of neutralizing anti-chimeric antibodies and functional PBMCs at the time of mAb therapy.

Monoclonal antibodies like Dintuximab beta are supposed not to penetrate the healthy blood–brain barrier (BBB). When drug levels of Db in cerebrospinal fluid were determined in three patients with acute neurotoxic side effects of the immunotherapy, no relevant levels were measured [26]. Actually, relapse after Db immunotherapy occurred in both of our patients intracranial. If this can be related to insufficient penetration of Db into the central nervous system or reflects common route of RB metastasis can´t be answered, especially with respect to GD2 negative relapse in patient 3. On the other hand, some findings indicate, that immunotherapeutic approaches with anti-GD2 antibodies can be a promising therapeutic option for GD2-expressing intracerebral or intraretinal tumors and metastases. Disrupted blood-retinal barrier in retinoblastomas [13] and the increased BBB-permeability caused by NB/RB metastasis and by irradiation, can allow effector cells and mAbs to exert effects in CNS tumors. Furthermore, the murine antibody 14.G2a was found to bind to the cerebellum [27], and the chimeric antibody m3F8 has been reported to cause disruption to the BBB itself [28].

Our retrospective analysis has several limitations. The cohort is very small without any control group. Patient 2 did not receive the full treatment of Db due to side effects. Patients received the immunotherapy following ASCT ± radiation and the effects of the different treatment modalities can´t be completely distinguished. Thus, the analysis does not allow to draw firm conclusions on the efficacy of the approach. However, anti-GD2-directed immunotherapy resulted in a response in both patients with evaluable residual tumor seen in MRI. These results, in addition to our in vitro assays, encourage further investigation in high-risk retinoblastoma, although prospective randomized studies are unlikely to occur due to the small number of expected eligible patients.

Four years after diagnosis of retinoblastoma and its treatment, including radiation, secondary alveolar Rhabdomyosarcoma (aRMS) developed in patient 2. Heterozygous pathogenic variants in RB1 in constitutional DNA represents not only a risk factor for the development of retinoblastoma but also other subsequent primary malignancies (SPM) particularly soft tissue sarcoma and osteosarcoma [29, 30]. Risk for secondary soft tissue sarcoma is even higher after external beam radiotherapy (EBRT) [29] and soft tissue sarcoma are more likely to develop on the head within the radiation field [31]. This effect was found to be dose dependent [30] and age related [32]. To account for these relations, the use of radiotherapy was confined in favor of chemotherapy [2]. Especially in those patients with hereditary retinoblastoma, GD2-directed immunotherapy might be of interest to substitute or delay radiotherapy.

Despite intense pretherapy in all patients, safety and tolerability of the immunotherapy was acceptable and comparable to the use of Dinutuximab beta in neuroblastoma patients [33]. Only in patient 2 therapy had to be terminated definitely due to anaphylaxis, which is a known common (rate from ≥ 1/100 to < 1/10) side effect [34].

Furthermore, no cumulative toxicity or carcinogenic potential of anti-GD2-directed immunotherapy has been reported and immunotherapy might be repeated in case GD2-positive metachronous tumors.

In patient 3 a GD2-negative relapse occurred. Loss of GD2 is a rare event in neuroblastoma following anti-GD2 immunotherapy [21, 35] and can be partly explained by reduced antigen production or by internalization of antigen/antibody complexes [36, 37]. In our patient, theoretically a separate GD2-negative retinoblastoma could have developed on the basis of the RB1 germline mutation, independent from the immunotherapy. Metachronous tumors and secondary malignancies are not uncommon in patients with hereditary retinoblastoma [38, 39]. Since both eyes were enucleated at this stage and no retinal tissue left, from which a new retinoblastoma could have developed, we regard this unlikely.

Thus, we suppose that in our patient a GD2 negative retinoblastoma occurred, which escaped the Dinutuximab therapy. Indeed, in vitro studies evaluating cytotoxicity of Anti-GD2 CAR T-cells in RB cell-lines showed a downregulation of GD2 [15]. However, if a loss of GD2 occurs more often in retinoblastoma than in neuroblastoma can´t be answered here. The escape of GD2 negative clones might be prevented by directing immunotherapy against different targets subsequently or in parallel. In case of GD2-negative tumors or GD2 loss, B7-H3 might serve as a potential alternative target. Several anti-B7-H3 therapies are currently investigated [40, 41].

Expression of B7-H3 both on RB cell lines and the tumor of patient 3 was shown. Using patient’s PBMCs and an anti-B7-H3 mAb, a clear ADCC effect against the RB cell line RBL-30 was demonstrated.

While survival for retinoblastoma in high-resource countries generally is high, there is a great global disparity [42]. Presentation in low-income countries (LIC) is usually at late stage [43, 44], the disease seems to be more aggressive and access to therapy is limited [45]. Estimated mortality of 40–80% [42, 45] highlights the need of alternative therapies in these countries. Despite anti-GD2 immunotherapy is currently high priced, the therapy might be feasible in low-income setting, particularly regarding less pronounced cytopenia.

In summary, we rate Anti-GD-2 immunotherapy a promising alternative in treatment of retinoblastoma patients. Especially in patient with disposition for retinoblastoma saving chemotherapy or radiation is most important due to their carcinogenic effects. Also, therapeutic options for advanced stages of retinoblastoma or relapsed disease are needed.

## Data Availability

The data that support the findings of this study are available from the corresponding author, TE, upon reasonable request.
